# Next generation chimeric antigen receptor T cells: safety strategies to overcome toxicity

**DOI:** 10.1186/s12943-019-1057-4

**Published:** 2019-08-20

**Authors:** Shengnan Yu, Ming Yi, Shuang Qin, Kongming Wu

**Affiliations:** Department of Oncology, Tongji Hospital of Tongji Medical College, Huazhong University of Science and Technology, 1095 Jiefang Avenue, Wuhan, 430030 People’s Republic of China

**Keywords:** Chimeric antigen receptor, Toxicity, Immunotherapy, Suicide gene, Synthetic notch receptor

## Abstract

Chimeric antigen receptor T (CAR-T) cell therapy is an emerging and effective cancer immunotherapy. Especially in hematological malignancies, CAR-T cells have achieved exciting results. Two Anti-CD19 CAR-T therapies have been approved for the treatment of CD19-positive leukemia or lymphoma. However, the application of CAR-T cells is obviously hampered by the adverse effects, such as cytokines release syndrome and on-target off-tumor toxicity. In some clinical trials, patients quitted the treatment of CAR-T cells due to life-threatening toxicity. Seeking to alleviate these toxicities or prevent the occurrence, researchers have developed a number of safety strategies of CAR-T cells, including suicide genes, synthetic Notch receptor, on-switch CAR, combinatorial target-antigen recognition, bispecific T cell engager and inhibitory CAR. This review summarized the preclinical studies and clinical trials of the safety strategies of CAR-T cells and their respective strengths and weaknesses.

## Introduction

Many studies have proven that immunity plays an essential role in the development of cancers [1, 2]. Therefore, immune therapies for malignant tumors including chimeric antigen receptor T (CAR-T) cells [3], bispecific antibodies [4], immune checkpoint inhibitors [5, 6], etc. have become research hotspots, and attracted the attention of more and more researchers and clinicians. In particular, as an adoptive cell therapy (ACT), CAR-based immunotherapy has achieved promising response [7, 8]. Patient-derived T cells are modified to express a CAR that is mainly composed of extracellular single-chain variable fragment (scFv) recognizing tumor antigens, transmembrane domain, intracellular immunoreceptor tyrosine-based activation motifs (ITAMs) from CD3 zeta chain (CD3ζ) and co-stimulatory domain [9]. The CAR-T cells recognize tumor antigens and are activated independent of major histocompatibility complex (MHC) [10]. In order to enhance the activity and persistence of CAR-T cells, researchers developed the second generation CAR containing one costimulatory domains (CD28 or 4-1BB or OX-40) and the third generation CAR containing two or more costimulatory domains on the basis of the first generation of CAR (no costimulatory domain) [11, 12]. The fourth generation CAR-T cells, also called TRUCKs, are engineered to secrete transgenic cytokine like interleukin-12 aiming at remodeling of tumor environment to promote therapeutic success [13, 14]. CAR-T cells have achieved remarkable clinical outcome in the application of malignant hematological tumors, such as acute lymphoblastic leukemia (ALL) [15, 16], chronic lymphocytic leukemia (CLL) [17, 18], and non-Hodgkin lymphoma (NHL) [19]. At present, two anti-CD19 CAR-T schemes have been approved by the US Food and Drug Administration (FDA). There are Novartis’s Kymriah for certain pediatric and young adult patients with a form of ALL and Gilead’s Yescarta for adult patients with relapsed or refractory large B-cell lymphoma [20]. Despite the high rate of remission in hematological malignancies, there is also a high rate of relapse which remains a major issue regarding the overall efficacy of CAR-T cells therapy. Due to the poor permeability, target selection and suppressive tumor microenvironment etc., the clinical outcome of CAR-T cells in solid tumors is less than that in hematological tumors [21, 22]. Although the current application of CAR-T cells has made some progress, the further development of CAR-T cells has been hindered with the serious side effects of CAR-T cells. After infused with CAR-T cells, patients usually suffer some adverse reactions, the most commons of which are cytokine release storm, tumor lysis syndrome, and on-target off-tumor toxicity [23]. In an attempt to reduce these adverse effects, researchers proposed a variety of safety strategies, including suicide genes, combinatorial target-antigen recognition, synthetic Notch receptors, on-switch CAR, and inhibitory CAR. Moreover, several approaches of alleviating toxicity of CAR-T cells have been entered clinical trials (shown in Table 1). Each safety strategy of CAR-T cells has a unique mechanism of action, so they have diverse strengths and weaknesses as summarized in Table 2.

**Table 1:** The clinical trials of next generation of CAR-T cells in cancer immunotherapy

Safety strategy	Target	Identifier	Disease	Treatment arms	Phase	Stage	Sponsor	Comments
EGFRt + cetuximab	CD19	NCT02028455	CD19^+^ acute leukemia	Anti-CD19 CAR-T/EGFRt	I/II	Recruiting	Seattle Children’s Hospital	To study the MTD and efficacy of CAR-T cells
		NCT02146924	High-risk ALL	Anti-CD19 CAR-T/EGFRt	I	Recruiting	City of Hope Medical Center	To study the side effects and best dose of CAR-T cells
		NCT01815749	Recurrent or high-risk NHL	Anti-CD19 CAR-T/EGFRt +auto-HSCT	I	Active, not recruiting	City of Hope Medical Center	To study the side effects and best dose of CAR-T cells
		NCT03579888	CD19^+^ lymphoid malignancies	Anti-CD19 CAR-T/EGFRt +Cyclophosphamide +Fludarabine	I	Not yet recruiting	M.D. Anderson Cancer Center	To study the side effects and best dose of CAR-T cells
		NCT02051257	Recurrent B-cell NHL	Anti-CD19 CAR-T/EGFRt	I	Active, not recruiting	City of Hope Medical Center	To study the highest dose of memory enriched T cells
		NCT01865617	R/R CLL, NHL or ALL	Anti-CD19 CAR-T/EGFRt	I/II	Recruiting	Fred Hutchinson Cancer Research Center	To study the side effects and best dose of CAR-T cells
		NCT03103971	R/R B-Cell NHL or ALL	Anti-CD19 CAR-T/EGFRt +Cyclophosphamide +Fludarabine	I	Recruiting	Fred Hutchinson Cancer Research Center	To study the side effects of CAR-T cells
		NCT03085173	R/R CLL	Anti-CD19 CAR-T/EGFRt	I	Recruiting	Memorial Sloan Kettering Cancer Center	To study the MTD of CAR-T cells
	CD123	NCT02159495	CD123^+^ R/R AML and persistent/recurrent BPDCN	Anti-CD123 CAR-T/EGFRt +Fludarabine	I	Recruiting	City of Hope Medical Center	To study the side effects and the best dose of CAR-T cells
		NCT03114670	Recurrent AML after allo-HSCT	Anti-CD123 CAR-T/EGFRt	I	Recruiting	Affiliated Hospital to Academy of Military Medical Sciences	To study the safety and effectiveness of CAR-T cells
	CD22	NCT03244306	CD22^+^ leukemia	Anti-CD22 CAR-T/EGFRt	I	Active, not recruiting	Seattle Children’s Hospital	To study the safety and feasibility of CAR-T cells
	CD171	NCT02311621	Neuroblastoma	Anti-CD171 CAR-T/EGFRt	I	Recruiting	Seattle Children’s Hospital	To study the MTD of CAR-T cells
	EGFR	NCT03618381	R/R solid tumors	Anti-EGFR CAR-T/EGFRt	I	Recruiting	Seattle Children’s Hospital	To study the safety, feasibility, and efficacy of CAR-T cells
	HER2	NCT03500991	HER2^+^ R/R pediatric CNS tumors	Anti-HER2 CAR-T/EGFRt	I	Recruiting	Seattle Children’s Hospital	To study the safety and feasibility of CAR-T cells via an indwelling CNS catheter
	BCMA	NCT03070327	Multiple myeloma	Anti-BCMA CAR-T/EGFRt +Cyclophosphamide +Lenalidomide	I	Recruiting	Memorial Sloan Kettering Cancer Center	To study the safety of CAR-T cells
iCasp9+ AP1903	GD2	NCT01822652	Neuroblastoma	Anti-GD2 CAR-T/iCasp9	I	Active, not recruiting	Baylor College of Medicine	To study the highest dose of CAR-T cells
		NCT01953900	GD2^+^ osteosarcoma neuroblastoma	Anti-GD2 CAR-T/iCasp9	I	Recruiting	Baylor College of Medicine	To study the largest safe dose of CAR-T cells
		NCT02107963	GD2^+^ solid tumors	Anti-GD2 CAR-T/iCasp9 +Cyclophosphamide	I	Completed	National Cancer Institute	No results posted
		NCT03721068	Neuroblastoma	Anti-GD2 CAR-T/IL-15/iCasp9 + Fludarabine +Cyclophosphamide	I	Recruiting	UNC Lineberger Comprehensive Cancer Center	To study the safety and feasibility of CAR-T cells
	CD19	NCT03016377	R/R ALL	Anti-CD19 CAR-T/iCasp9 +Cyclophosphamide +Fludarabine	I	Recruiting	UNC Lineberger Comprehensive Cancer Center	To study the safety and feasibility of CAR-T cells, and the optimal dose of AP1903
		NCT03696784	R/R B-cell lymphoma	Anti-CD19 CAR-T/iCasp9 + Bendamustine +Fludarabine	I	Not yet recruiting	UNC Lineberger Comprehensive Cancer Center	To study the safety and feasibility of CAR-T cells
	CD19 CD22	NCT03098355	B-cell leukemia and lymphoma	Anti-CD19/22 CAR-T/iCasp9 + IL-2	I/II	Recruiting	Zhujiang Hospital	To study the safety, efficacy and persistence of CAR-T cells
	CD30	NCT02274584	CD30^+^ lymphomas	Anti-CD30 CAR-T/iCasp9	I/II	Recruiting	Peking University	To study the safety of CAR-T cells
	Mesothelin	NCT02414269	Mesothelioma, lung cancer or breast cancer	Anti-mesothelin CAR-T/iCasp9 + Cyclophosphamide	I	Recruiting	Memorial Sloan Kettering Cancer Center	To study the safety of CAR-T cells
TanCAR	CD19 CD20	NCT03019055	R/R CD19^+^ or CD20^+^ B-cell malignancies	Anti-CD19/20 CAR-T cells	I/II	Recruiting	Medical College of Wisconsin	To study the safety and feasibility of CAR-T cells
		NCT03097770	R/R B-cell leukemias and lymphomas	Anti-CD19/20-CAR-T cells	NA	Recruiting	Chinese PLA General Hospital	To study the safety and feasibility, and the duration of in vivo survival of tanCART-19/20 cells

EGFRt: truncated epidermal growth factor receptor; MTD: maximum tolerated dose; CNS: central nervous system; AML: acute myeloid leukemia; BPDCN: blastic plasmacytoid dendritic cell neoplasm; Allo-HSCT: allogeneic hematopoietic stem cell transplantation; Auto-HSCT: autologous hematopoietic stem cell transplantation; ALL: acute lymphoblastic leukemia; NHL: non-Hodgkin’s lymphoma; CLL: chronic lymphoblastic leukemia; R/R: relapsed/refractory; NA: not applicable

**Table 2:** The strengths and weaknesses of various safety strategies of CAR-T cells

Safety strategy		Strengths	Weaknesses
Suicide switch	HSV-TK	1. powerful effect 2. extensive clinical experience	1. immunogenicity 2. clinical incompatibility 3. slow onset 4. no preventive effect for toxicity 5. premature eradication of CAR-T cells
	iCasp9	1. no immunogenicity 2.clinical compatibility 2. rapid onset	1. no preventive effect for toxicity 2. premature eradication of CAR-T cells
	CD20	1. no immunogenicity 2. rapid onset	1. antibody biodistribution 2. on-target toxicity from antibody 3. prodrug infusion reaction 4. no preventive effect for toxicity 5. premature eradication of CAR-T cells
	EGFRt	1. no immunogenicity 2. rapid onset 3. in vivo tracking	1. antibody biodistribution 2. on-target toxicity from antibody 3. prodrug infusion reaction 4. no preventive effect for toxicity 5. premature eradication of CAR-T cells
Endogenous switch	synNotch	1. control the expression of the CARs 2. Specific recognition of tumor sites	1. uncontrolled activation of CAR-T cells 2. the choice of two antigens is difficult
	iCAR	1. antigen-selectively regulate T cell responses 2. protect normal tissue from CAR-T cells	1. uncontrolled activation of CAR-T cells 2. potential “on-target, off-tumor” effect
	Combinatorial Target-Antigen Recognition	1. precise killing of CAR-T cells 2. overcome antigen loss	1. uncontrolled activation of CAR-T cells 2. the choice of two antigens is difficult 3. potential “on-target, off-tumor” effect
Exogenous switch	Bispecific T Cell Engager	1. controlled activation of CAR-T cells 2. simplify manufacturing of CAR-T cells	1. The choice of small molecules needs more consideration
	On-switch CAR	1. controlled activation of CAR-T cells	1. The choice of small molecules needs more consideration

## Adverse effects of CAR-T cells

### On-target on-tumor toxicity

The cytokine release syndrome (CRS) is the most common toxicity of CAR-T cells due to the excessive cytokine release [24]. When CAR-T cells are activated by the corresponding target cells, they release a large number of cytokines. In addition to killing tumor cells, these excessive cytokines cause various clinical symptoms, including fever, tachycardia, hypotension and hypoxia, potentially leading to rapid death [25]. As previously reported that the levels of several cytokines including tumor necrosis factor-alpha (TNF-α), interferon γ (IFN-γ), interleukin 6 (IL-6), and IL-10 are markedly elevated in patient serum after receiving CAR-T cells [26]. Some biomarkers, such as CAR-T cells dosage, disease burden, have been applied to predict patients’ risk suffering severe CRS during CAR-T cell therapy [27]. Grading CRS can be useful to guide the management of severe CRS [28, 29]. Furthermore, tumor lysis syndrome (TLS) is another common toxicity, especially in hematological tumors, with overlap in the symptoms of CRS. Due to the destruction of large amount of tumor cells, the rapid release of intracellular substances lead to a series of metabolic disorders such as hyperuricemia, hyperkalemia, hyperphosphatemia, hypocalcemia and metabolic acidosis, which lead to life-threatening severe arrhythmia or acute renal failure [30]. In addition to administrate high-dose corticosteroids or corresponding cytokine inhibitors (such as IL-6 receptor antagonist mAb, tocilizumab), reducing the number of CAR-T cells or using switchable CAR-T cells also mitigate these clinical symptoms [31].

### On-target off-tumor toxicity

Because there are few available tumor-specific antigens (TSAs), the targets that recognized by CAR-T cells are always tumor-associated antigens (TAAs), which are weakly expressed in normal tissues. Therefore, the on-target off-tumor toxicity is an unavoidable side effect. CAR-T cells have achieved promising results in hematological tumors. However, since the target antigens (CD19, CD20, CD22) are also expressed on some normal blood cells, on-target off-tumor toxicity such as B-cell aplasia become a major obstacle for application of CAR-T cells in hematological tumors [16]. In solid tumors, similar phenomena exist. As previously reported that a patient with colon cancer metastatic to the lungs and liver, experienced respiratory distress within 15 min after anti-ERBB2 CAR-T cells infusion. Finally, the patient still failed to survive 5 days after treatment. The final conclusion was that CAR-T cells recognized low levels of ERBB2 on lung epithelial cells [32].

### Other adverse effects of CAR-T cells

Allogeneic hematopoietic stem cell transplantation (allo-HSCT) is an important treatment strategy for hematological malignancies. However, the success of allo-HSCT may be hindered by graft-versus-host disease (GVHD). Donor-derived CAR-T cells may increase the risk of GVHD occurrence. Ghosh et al. demonstrated that CAR-T cells with cumulative TCR and CAR signaling could reduce the risk of GVHD [33]. Most antigen-recognition domains of CARs derived from murine antibodies which may cause host anti-CAR response [34]. A study reported that T cells were armed with a CAR derived from a murine mAb against human mesothelin resulted in acute anaphylaxis due to the immunogenicity of infusion foreign proteins. Especially the transient efficacy of CAR-T cells requires repeated infusion which leads to the production of IgE antibodies [35]. Long-lived CAR-T cells or fully humanized antibodies may eliminate the anaphylaxis and immunogenicity. T cells transfected with viral vectors to express antigen-specific CARs may pose a potential risk of oncogenic insertional mutagenesis. Although there was no evidence of viral vector-induced immortalization of cells, it should be noted that genotoxicity may occur in the clinical application of CAR-T cells, especially in long-term monitoring [36]. In addition, neurotoxicity was also observed during the treatment of CAR-T cells, which generally included confusion, delirium, expressive aphasia, obtundation, myoclonus, and seizure [8, 15]. After infusion of anti-CD19 CAR-T cells, the modified T cells were detectable in the blood, bone marrow, and cerebrospinal fluid (CSF) of patients with neurotoxicity [7]. Furthermore, another study showed that cytokine levels including IFN-γ and IL-6 in CSF were extremely higher than those in the serum. CAR-T cells penetrate the blood-brain barrier (BBB) and produce a large number of cytokines, causing cerebral CRS [37]. Because tocilizumab or other monoclonal antibodies are incapable of passing the BBB, corticosteroid is the drug of choice for the management of cerebral CRS [38].

## Safety strategies to overcome the toxicity of CAR-T cells

### Suicide gene switch

**Fig. 1:**
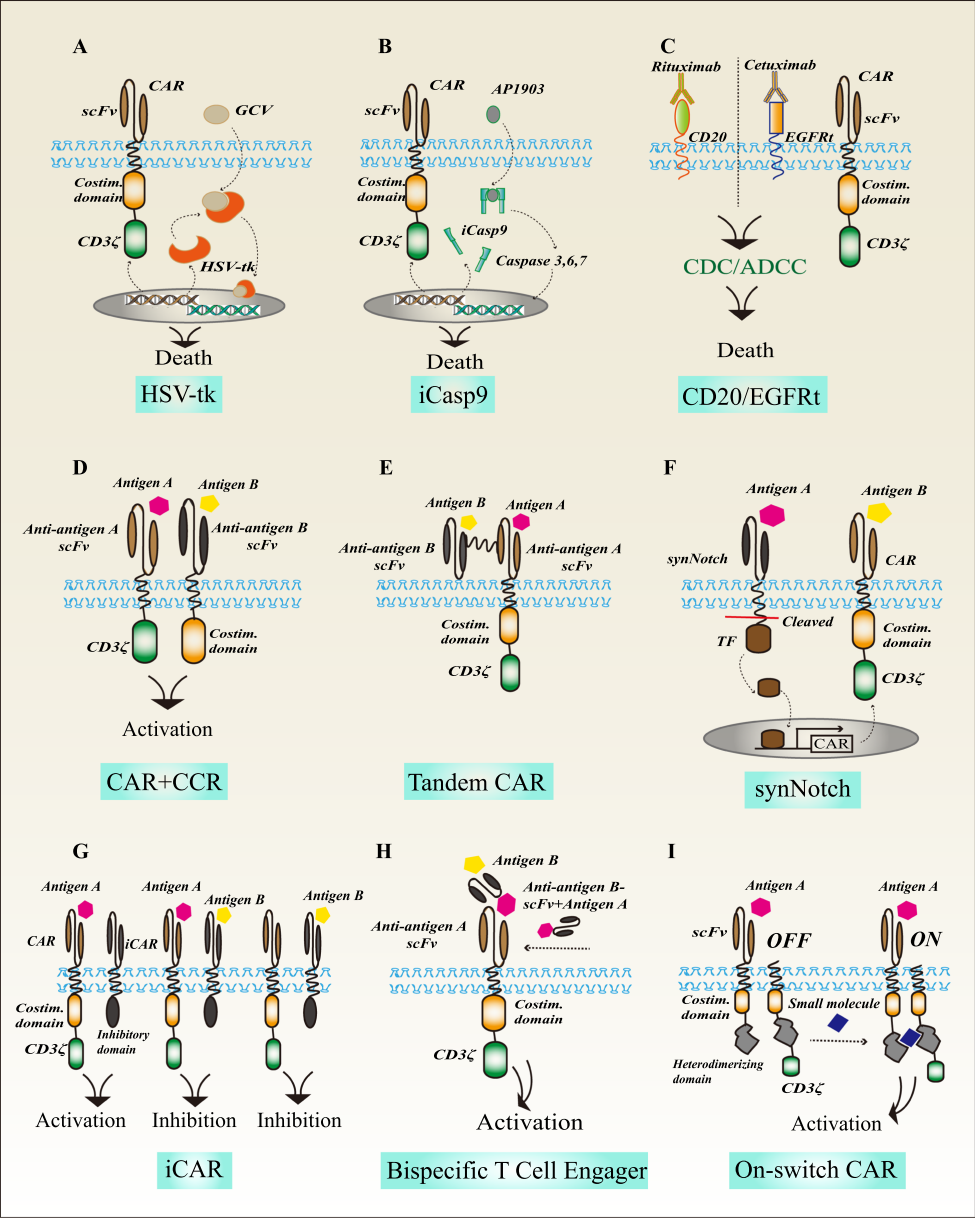
Summarized safety strategies for CAR-T cells to overcome toxicity. **a** HSV-tk phosphorylates GCV, forming toxic GCV-triphosphate compound that competes with triphosphate as a substrate incorporated into DNA, leading to inhibition of DNA synthesis and the cellular death. **b** Conditional administration of AP1903 forms dimerization with iCasp9 and activates the downstream caspase molecules, resulting in apoptosis of CAR-T cells. **c** CAR-T cells expressing CD20 or EGFRt could be efficiently and specifically eliminated with clinically approved monoclonal antibody rituximab or cetuximab through CDC/ADCC. **d** T cells were transduced a CAR with CD3ζ recognizing one antigen and a CCR with CD28 and/or 4-1BB binding another antigen. **e** TanCAR comprised of two tandemly linked scFvs targeting different tumor antigens coupled with one activation domain. **f** The synNotch receptor first recognized a tumor antigen, and then leading to the release of a transcriptional activator domain to drive the expression of a CAR that targeting another tumor antigen. **g** The iCAR consists of a scFv specific to the antigens expressed exclusively on normal tissue, and an inhibitory signaling domain of immunoinhibitory receptors (PD-1 and CTLA-4) to restrict T cell activity despite concurrent engagement of an activating receptors. **h** CAR-T cells do not directly recognize antigen on target cells, but they are recruited to effector cells through a bispecific small molecule. **i** On-switch CAR-T cells are activated by antigen recognition and a small molecule to connect the costimulatory domains and the splitting downstream ITAMs

Herpes simplex virus thymidine kinase (HSV-tk) is the best characterized suicide gene [39] and widely used in combination with ganciclovir (GCV) for the treatment of a variety of malignant cancers [40, 41]. HSV-tk phosphorylates specific nucleoside analogues, such as GCV, forming toxic GCV-triphosphate compound that competes with triphosphate as a substrate incorporated into DNA via the action of DNA polymerase, leading to the inhibition of DNA synthesis and subsequent cellular death [42, 43] (Fig. 1a). The most extensive application of the HSV-tk suicide gene is to eliminate GVHD in the setting of donor lymphocyte infusion after hematopoietic stem cell transplantation (HSCT) [44, 45]. Donor T lymphocytes expressing HSV-tk suicide gene were infused into patients with leukemia after haploidentical hematopoietic stem cell transplantation (haplo-HSCT) to facilitate immune reconstitution and control GVHD [46, 47]. HSV-tk suicide gene was also applicated in CAR-T cells targeting CD44 isoform variant 6 (CD44v6), which is overexpressed in acute myeloid leukemia (AML) and multiple myeloma (MM). The pre-clinical study indicated that anti-CD44v6 CAR-T cells expressing HSV-tk showed potent antitumor efficacy and could be effectively eliminated upon exposure to GCV [48]. Although the efficacy and feasibility of HSV-tk have been validated, there are still several limitations of HSV-tk: (1) HSV-tk is derived from virus with potential immunogenicity, therefore it may compromise the survival of functionally modified T cells [49]; (2) HSV-tk requires activation by a prodrug (like GCV) that remains a crucial pharmacologic agent for the treatment of cytomegalovirus infection [50]; (3) Because interfering DNA synthesis to induce T cells death is a gradual progress, it takes more time to eliminate CAR-T cells by HSV-tk [47].

The inducible safety switch caspase 9 (iCasp9) suicide gene contains a modified human caspase 9 fused to the human FK506 binding protein (FKBP). Conditional administration of a chemical inducer of dimerization (CID) (AP1903) forms dimerization and activates the downstream caspase molecules, resulting in apoptosis of cells expressing the fusion protein [51] (Fig. 1b). The ultimate goal of CAR-T therapy is always efficacy and safety. A second generation CAR targeting the CD19 antigen co-expressed with IL-15 and the inducible suicide gene caspase 9 (iCasp9/CAR-CD19/IL-15). IL-15 is a cytokine critical for T cells expansion and survival [52]. The iCasp9/CAR-CD19/IL-15 T cells showed superior survival, expansion and anti-tumor activity in vivo and in vitro compared to only CAR-CD19 T cells. And more importantly, the incorporation of iCasp9 suicide gene and its pharmacologic activation efficiently eliminated these gene engineered T cells. The administration of iCasp9 suicide gene further increased the safety of the proposed approach and its potential clinical applicability [53]. A third generation anti-CD20 CAR-T cells containing both CD28 and 4-1BB co-stimulatory domains were transduced with iCasp9 suicide gene and a truncated CD19 (ΔCD19) selectable marker. The iCasp9/CAR-CD20/ΔCD19 T cells caused effective cytotoxicity to CD20-positive tumor cells and induced potent cytokines secretion. 20 nM AP1903 eliminated 90% of the transduced T cells within 24 h and 98% were dead after 72 h. In addition, the CID treatment has no impact on the growth of non-transduced T cells even at concentrations as high as 200 nM [54]. Long-term B cell aplasia is one of the manifestations of “on-target off-tumor” toxicity for the application of anti-CD19 CAR-T cells in B cell lymphoid malignancies, which strongly correlated with long-term survival [55]. The anti-CD19 CAR-T cells incorporated with iCasp9 suicide gene and a truncated nerve growth factor receptor (ΔNGFR) as selective marker exhibited potent cytotoxic activity against CD19-positive tumor cells in vitro. In a humanized mouse model, the iCasp9 safety switch efficiently eliminated anti-CD19 CAR-T cells in a dose-dependent manner, allowing normal B cell reconstitution [56]. CD33 is overexpressed on the cell surface of 90% of AML blasts [57]. However, it is also expressed on multipotent myeloid precursors [58]. iCasp9 suicide gene and ΔCD19 modified CD33 CAR-T cells (iCasp9/CAR CD33/ΔCD19) not only are cytotoxic to CD33-positive tumor cells but also could be used as a “bridge” therapy for patients coming to allo-HSCT. Due to a proportion of the infused CD34^+^ hematopoietic stem cells also express CD33, it is essential to completely eliminate the CD33 CAR-T cells prior to stem cell infusion to reduce the potential risk of engraftment failure. The conditional administration of CID resulted in the elimination of only 76.4% anti-CD33 CAR-T cells [59]. Previous study demonstrated that iCasp9 resistant cells expressed higher anti-apoptotic molecule B-cell lymphoma 2 (BCL-2) [60]. Therefore, the co-administration of suicide gene/CID and BCL-2 inhibitor resulted in additive effect up to completely cell elimination [59]. Another iCasp9 engineered CAR-T cells targeting interleukin-1 receptor accessory protein (IL-1RAP) which is expressed by chronic myeloid leukemia (CML) but not the normal CD34^+^ hematopoietic stem cell [61] showed cytotoxicity against IL-1RAP-positive cell lines or primary CML cells and induced secretion of pro-inflammatory cytokines in vitro and in vivo. In the NSG murine model, 87% of anti-IL-1RAP CAR-T cells were eliminated after intraperitoneal injection of AP1903, but control T cells were not affected [62]. iCasp9 suicide gene as a T-cell safety switch also has been entered clinical trials. Haplo-HSCT is an alternative therapeutic strategy for patients without a more closely matched donor or who need an urgent allo-HSCT [63]. However, patients are more prone to occur GVHD after transplantation, mainly due to the existence of the non-shared HLA haplotype [64]. Adoptive transfer of donor derived engineer iCasp9-T cells may promote immune reconstitution and reduce the risk of uncontrolled GVHD. In a phase I trial (NCT00710892), genetically modified iCasp9/ΔCD19 T cells were infused into five patients that had undergone haploidentical transplantation for relapsed acute leukemia. Four patients developed into GVHD after the infusion of allodepleted T cells were treated with a single dose (0.4 mg/kg) of AP1903. More than 90% transgenic T cells were eliminated within 30 min after administration of AP1903. GVHD-associated manifestations of skin and liver started to alleviate within 24 h [65]. Long-term follow-up (more than 2 years) results showed that the iCasp9 suicide gene not only permanently controlled GVHD, but also contributed to improve recovery of immune T cell to control opportunistic infections after haplo-HSCT [66]. In addition, another similar clinical trial (NCT01494103) results suggested that iCasp9-T cells reconstituted immunity posttransplant and provided protection from EBV, CMV, HHV6, VZV and BKV infections. Administration of AP1903 eliminated iCasp9-T cells from peripheral blood (PB) and the central nervous system (CNS) leading to the rapid remission of GVHD and CRS [67]. The humanized iCasp9 suicide gene did not caused immunogenicity, thus avoiding the attack from host immunity to engineered CAR-T cells. Moreover, non-toxic exogenous small molecule drug and rapid induction of apoptosis make iCasp9 to be a better option of safety method of CAR-T cells.

T cells modified to co-express the CAR and CD20 or truncated epidermal growth factor receptor (EGFRt) could be efficiently and specifically eliminated with clinically approved monoclonal antibody rituximab or cetuximab through the complement dependent cytotoxicity (CDC) and antibody-dependent cell-mediated cytotoxicity (ADCC) [68, 69] (Fig. 1c). In particular, EGFRt-rituximab suicide switch has been incorporated into the CAR-T cells to mitigate toxicity in numerous clinical trials, which were summarized in Table 1. Although antibody-mediated suicide switch showed significant effects in controlling the toxicity of engineered CAR-T cells in preclinical and clinical studies [70, 71], the biodistribution of therapeutic antibodies and possible damage to normal tissues expressing the specific proteins may limit the further development [72].

### Combinatorial target-antigen recognition

Dividing the traditional CAR into two complementary parts may be a promising approach to enhance safety. T cells were transduced a CAR with CD3ζ recognizing one antigen and a chimeric costimulatory receptor (CCR) with CD28 and/or 4-1BB binding another antigen (Fig. 1d). Based on the strategy, combinatorial using the prostate-specific membrane antigen (PSMA) and prostate stem cell antigen (PSCA), Klossetal et al. developed the co-transduced CAR-T cells and demonstrated that the T cells efficiently eliminated tumors that express both antigens but did not affect tumors expressing either antigen alone. Moreover, their results showed that T cells expressing suboptimal activation CAR (low affinity) and a CCR are sufficiently activated by PSMA^+^PSCA^+^ but not PSMA^+^PSCA^−^ target cells. However, when T cells transduced efficient activation CAR (high affinity) and a CCR recirculate, they could kill the PSMA^+^PSCA^−^ target cells without requiring further costimulation, resulting in an “on-target off-tumor” adverse effect on normal tissues [73]. Another study reported a combinatorial target-antigen recognition CAR strategy that activation receptor with CD3ζ against mesothelin is physically dissociated from CCR with CD28 against a-folate receptor (FRa). The dual-targeting CAR-T cells exhibited significant cytotoxicity to ovarian cancer cells expressing two antigens in vitro and in pre-clinical animal studies and coupled with minimized damage to normal tissues [74]. Wilkie et al. also designed the “dual-targeted” T cells co-expressing an ERBB2- and MUC1-specific CAR that signal using CD3ζ and CD28 respectively. However, these dual CAR-T cells only eliminated ERBB2^+^ tumor cells, and produced modest IL-2 in response to co-stimulation with a second antigen when compared to a control CAR-T cells expressing CD28 and CD3ζ endodomain [75]. In addition, two tandem linked scFvs targeting different tumor antigens coupled with one activation domain forming a CAR called tandem CAR (TanCAR) [76] (Fig. 1e). Studies demonstrated that TanCAR T cells exhibited significant cytotoxicity to glioblastoma (HER2/IL13Rα) and leukemia (CD19/CD133) in vitro and in vivo [77, 78]. T cells expressing TanCAR simultaneously recognized two different antigens on tumor cells, which reduced antigen escape and enhanced antitumor efficacy, as well as mitigated “on-target off-tumor” toxicity [79, 80]. However, it is highly dependent on the two targeted antigens that uniformly expressed in a particular cancer type, but expressed differentially and at low levels in normal tissues.

### Synthetic notch receptors

A recently study reported a novel class of modular receptors called synthetic Notch (synNotch) receptors [81]. Wild-type Notch protein contains three parts: an extracellular ligand-binding domain, a transmembrane domain, releasing transcriptional regulator through ligand-induced cleavage, and the intracellular effector domain [82]. The extracellular recognition domain could be customized with antibody-based domain, such as single-chain antibody or nanobodies, to recognize specific antigens. Moreover, the intracellular transcription domain could be replaced to activate downstream interesting target genes. Therefore, the modified synNotch receptors only retain the core portion of the transmembrane region of wild-type Notch, which mediates proteolysis [81]. Based on the mechanism, Roybal et al. designed the synNotch AND-gate circuits for the safety of CAR-T cells. The synNotch receptor first recognized a tumor antigen, and then leading to the release of a transcriptional activator domain to drive the expression of a CAR that targeting another tumor antigen (Fig. 1f). Jurkat T cells engineering with synNotch receptor recognized CD19 and mesothelin effectively killed tumor cells that expressing two antigens but not one of these antigens. The synNotch AND-gate was tested in the primary T cells binding green fluorescent protein (GFP) and a CAR against CD19. The results indicated that the synNotch AND-gate T cells could selectively clear the dual antigens tumor in vivo, while avoiding the by-stander tissues [83]. Receptor tyrosine kinase-like orphan receptor 1 (ROR1) is expressed by many epithelial tumors and stromal cells. Targeting ROR1 not only will lyse ROR1+ tumor cells, but also cause toxicity on the ROR1+ normal tissues [84]. Targeting ROR1 CAR-T cells are engineered with synNotch receptors specific for EPCAM or B7-H3. The CAR-T cells showed selective cytotoxicity to ROR1+ tumor cells, but not ROR1+ stromal cells. According to the results, B7-H3 is a more suitable synNotch target due to the more frequent co-expression with ROR1 on cancers than EPCAM [85]. In addition, SynNotch T cells could be engineered to deliver cytokines, antibodies, or small molecules in response to antigens and remodel local microenvironments in a very precise and localized way [86].

### Inhibitory chimeric antigen receptor

Immune inhibitory receptors, such as PD-1, CTLA-4, play a crucial role in the regulation of immune response, especially in attenuating or terminating T cells response [87]. It is a promising approach that applying inhibitory receptors to CAR-T cells as a safety strategy. Fedorov et al. designed the PD-1- and CTLA-4-based inhibitory chimeric antigen receptor (iCAR) targeting PSMA. The iCAR consists of a scFv specific to the antigens expressed exclusively on normal tissue, and a powerful acute inhibitory signaling domain of immunoinhibitory receptors (PD-1 and CTLA-4) to restrict T cell activity despite concurrent engagement of an activating receptors (Fig. 1g). The iCAR showed antigen-specific suppression of T cell cytokine secretion, cytotoxicity, and proliferation in a temporary and reversible manner [88]. iCAR provides a dynamic, self-regulating safety switch to prevent the on target off-tumor side effects. However, iCAR T cells need to recognize tissue-specific antigens that are absent or down-regulated on tumors but expressed by the off-target tissues. And iCAR T cells cannot control their spatiotemporal activity.

### Bispecific T cell engager

T cells were engineered to express a CAR that binds a fluorescein isothiocyanate (FITC) molecule, termed “universal” anti-FITC–directed CAR-T cell. These CAR-T cells do not directly recognize antigen on target cells, but they are recruited to effector cells through a bispecific small molecule (Fig. 1h). Therefore, in the absence of bispecific small molecules, these CAR-T cells are inactive against normal tissues. The folate-FITC conjugate, as a bispecific small molecule switch, redirects and regulates anti-FITC-CAR-T cells activity to target folate receptor positive tumor cells in a specific and dose-dependent manner [89]. Various antitumor antibodies (Ab), such as cetuximab (Ctx; anti-EGFR), trastuzumab (anti-Her2), and rituximab (Rtx; anti-CD20), were conjugated with FITC forming bispecific small molecule Ab-FITC, which redirected anti-FITC-CAR-T cells binding to tumor cells and induced antitumor activity in vitro and in vivo [90]. Additionally, the antitumor effects of anti-FITC-CAR-T cells also confirmed in CD19- and CD22-expressing cancer cells. The Ab-FITC selectively led CAR-T cells to target cells and promoted the cytokine secretion that correlated with the degree of cytotoxicity [91]. Rodgers et al. developed the recombinant antibody-based bifunctional switches that consists of a specific anti-tumor antigen Fab molecule conjugated with a peptide neo-epitope (PNE) and a peptide-specific switchable CAR-T (sCAR-T) cell. The study indicated that sCAR-T cells provided equal lysis efficacy and lower cytokine levels compared to conventional anti-CD19 CAR-T cells in the presence of anti-CD19 Fab-PNE. Similarly, sCAR-T cells together with anti-CD20 Fab-PNE are suitable to eradicate CD20-positive tumor cells through the selective formation of immunological synapses [91]. T cells were modified to express a chimeric receptor CD16V-BB-ζ that included the high-affinity CD16 (FCGR3A) V158 variant, CD8a hinge, and transmembrane domains, as well as CD3ζ and 4-1BB. The CD16V-BB-ζ T cells recognized Fc fragment of specific antibody that targeting tumor antigens and prompted the capacity to exert ADCC. Antibody binding to the CD16V-BB-ζ receptor triggered T-cell activation, proliferation and specific cytotoxicity against target cells. Moreover, cytotoxicity was completely relied on the presence of a specific antibody binding to target cells. Dissociating antibodies neither provoke nonspecific cytotoxicity nor affected specific cytotoxicity [92]. The activity of anti-tag CAR-T cells could be attenuated by reducing the administration of bispecific small molecule when the adverse effects occur. Therefore, the bispecific T cell engager is used as a safety strategy of CAR-T cells due to the controllability.

### On-switch CAR

On-switch CAR consists of an extracellular specific antigen-binding domain (scFv) with costimulatory domains and a key downstream signaling element: the ITAMs from the T cell receptor CD3ζ subunit [93] (Fig. 1i). Recognition of the cognate antigen and application of a priming small molecule trigger the therapeutic activity of engineered-T cells, neither small molecule nor antigen should activate it alone. The small molecule-dependent on-switch approach precisely control the timing, location, and dosage of T cell activity in a titratable and reversible manner, thereby alleviating toxicity [94]. Juillerat et al. developed a “transient CAR-T cell” strategy, which dimerized at the hinge domain with the addition of a small molecule, thereby endowed the state of CAR-T cells from off to on. The engineered CAR-T cells presented a significant cytolytic activity only in presence of the AP21967, and reaction intensity was dependent on the amount of small molecule [95].

### Other strategies

In addition to the above superior safety strategies, other methods may also reduce the side effects of CAR-T cells. Regional delivery or intratumoral injection of CAR-T cells could be considered as a measure of reducing “off-tumor on-target” toxicity and enhancing antitumor efficacy [96–98]. A large number of activated CAR-T cells and lysed tumor cells will cause CRS and TLS. Transient CAR expression may be an available precaution to reduce these toxicities, which avoids the excessive reactivity of CAR-T cells [99]. Engineered T cells expressing a fully human CAR targeting the human C4 folate receptor-alpha (αFR) with an intermediate affinity can efficiently eliminate αFR-expressing tumors in vitro and in vivo. The CAR-T cells may overcome issues of transgene immunogenicity and less recognition of normal cells expressing low levels of αFR that mitigate “on-target off-tumor” toxicity [100]. CAR-T cells with decreased affinity to ErbB2 could discriminate tumor cells with high and low level ErbB2 from normal cells with physiologic level ErbB2 [101]. Other studies also demonstrated that low-affinity CAR-T cells targeting EGFR or CD123 could distinguish malignant from normal cells and reduce the “off-tumor on-target” toxicity [102, 103]. Moreover, some symptomatic treatments, such as cytokine-blocking agents and high-dose corticosteroids have also played a role in reducing the adverse effects of CAR-T cells [104, 105].

## Conclusion and perspectives

Adoptive cellular therapy with the engineered CAR-T cells is a promising approach for cancer treatment. And encouraging results have been achieved in hematological malignancies. But the toxicity of CAR-T cells has been a challenge and impedes the further development. Nowadays, numerous safety measures to overcome these adverse effects of CAR-T cells have been investigated in preclinical and clinical trials. Traditional suicide genes can quickly and effectively mitigate toxicity, but they also irreversibly eliminate therapeutic CAR-T cells which are expensive labor-intensive. Endogenous switches including synNotch, iCAR and CCR can cause intracellular regulation in a self-switch manner when CAR-T cells recognize responding antigens, which significantly reduce on-target off-tumor effects. However, these methods cannot control the timing and intensity of CAR-T cells activity. Bispecific T cell engager and on-switch CAR could achieve it by exogenously administrating small molecules. Of course, the exogenous small molecules should be safe and bio-inert, as well as have good pharmacokinetic properties. Although these strategies have potential shortcomings and need improvement or optimized new methods, we believe that the next generation CAR-T cells with safety switch could exhibit superior safety and efficacy, as well as bring more hopes to patients with malignant tumors.

## Data Availability

Data sharing not applicable to this article as no datasets were generated or analyzed during the current study.

## Abbreviations

ACT: adoptive cell therapy
ADCC: antibody-dependent cell-mediated cytotoxicity
ALL: acute lymphoblastic leukemia
allo-HSCT: allogeneic hematopoietic stem cell transplantation
AML: acute myeloid leukemia
BBB: blood-brain barrier
BCL-2: B-cell lymphoma 2
CAR-T: chimeric antigen receptor T
CCR: chimeric costimulatory receptor
CD3ζ: CD3 zeta chain
CD44v6: CD44 isoform variant 6
CDC: complement dependent cytotoxicity
CID: chemical inducer of dimerization
CLL: chronic lymphocytic leukemia
CML: chronic myeloid leukemia
CNS: central nervous system
CRS: cytokine release syndrome
CSF: cerebrospinal fluid
EGFRt: truncated epidermal growth factor receptor
FDA: Food and Drug Administration
FITC: fluorescein isothiocyanate
FKBP: FK506 binding protein
FRa: a-folate receptor
GCV: ganciclovir
GVHD: graft-versus-host disease
haplo-HSCT: haploidentical hematopoietic stem cell transplantation
HSV-tk: herpes simplex virus thymidine kinase
iCAR: inhibitory chimeric antigen receptor
iCasp9: inducible caspase 9
IFN-γ: interferon γ
IL-1RAP: interleukin-1 receptor accessory protein
IL-6: interleukin 6
ITAMs: immunoreceptor tyrosine-based activation motifs
MHC: major histocompatibility complex
MM: multiple myeloma
NHL: non-Hodgkin lymphoma
PB: peripheral blood
PNE: peptide neo-epitope
PSCA: prostate stem cell antigen
PSMA: prostate-specific membrane antigen
ROR1: receptor tyrosine kinase-like orphan receptor 1
sCAR-T: switchable CAR-T
scFv: single-chain variable fragment
synNotch: synthetic Notch
TAAs: tumor-associated antigens
TanCAR: tandem CAR
TLS: tumor lysis syndrome
TNF-α: tumor necrosis factor-alpha
TSAs: tumor-specific antigens
ΔNGFR: truncated nerve growth factor receptor
